# A Deep Learning Framework for Multi-Object Tracking in Space Animal Behavior Studies

**DOI:** 10.3390/ani15162448

**Published:** 2025-08-20

**Authors:** Zhuang Zhou, Shengyang Li, Yixuan Lv, Kang Liu, Yuxuan Cao, Shicheng Guo

**Affiliations:** 1 Technology and Engineering Center for Space Utilization, Chinese Academy of Sciences, Beijing 100094, China; zhouzhuang@csu.ac.cn (Z.Z.); lvyixuan@csu.ac.cn (Y.L.); liukang@csu.ac.cn (K.L.); caoyuxuan23@mails.ucas.ac.cn (Y.C.); guoshicheng@csu.ac.cn (S.G.); 2 Key Laboratory of Space Utilization, Chinese Academy of Sciences, Beijing 100094, China; 3 University of Chinese Academy of Sciences, Beijing 100049, China

**Keywords:** multi-object tracking, deep learning, space animal, spatio-temporal fusion

## Abstract

In space environments, microgravity, high radiation, and weak magnetic fields induce behavioral alterations in animals, resulting in erratic movement patterns that complicate tracking. These challenges impede accurate behavioral analysis, especially in multi-object scenarios. To address this issue, this study proposes a deep learning-based multi-object tracking (MOT) framework specifically designed for space animals. The proposed method decouples appearance and motion features through dual-stream inputs and employs modality-specific encoders (MSEs), which are fused via a heterogeneous graph network to model cross-modal spatio-temporal relationships. Additionally, an object re-detection module is integrated to maintain identity continuity during occlusions or rapid movements. This approach is validated using public datasets of space-observed *Drosophila* and zebrafish, with experimental results demonstrating superior performance compared with existing tracking methods. This work highlights the potential of artificial intelligence as a valuable tool in behavioral studies, enabling reliable animal tracking and analysis under extreme space conditions and supporting future research in space life sciences.

## Simple Summary

This study presents a deep learning-based multi-object tracking framework tailored for tracking animals in space environments, addressing challenges like microgravity-induced erratic movements, frequent occlusions, and high visual similarity among individuals. The framework decouples appearance and motion features via dual-stream inputs; employs modality-specific encoders fused through a heterogeneous graph network to model cross-modal spatio-temporal relationships; and integrates an object re-detection module for identity continuity during occlusions or rapid movements. Validated on public datasets of space-observed *Drosophila* and zebrafish, it outperforms existing methods, highlighting artificial intelligence’s potential in behavioral studies under extreme space conditions to support space life sciences research.

## 1. Introduction

Space-based life science experiments are increasingly critical for elucidating biological adaptation mechanisms in altered gravity environments and advancing innovative biotechnologies [1,2]. NASA’s Open Science platforms provide access to extensive behavioral datasets from rodent studies conducted aboard the International Space Station (ISS) [3], with recent analyses demonstrating that habitat factors such as elevated microgravity significantly alter movement patterns and activity rhythms—findings that inform the optimization of future animal housing and mission design. Parallel efforts by Japan’s National Space Development Agency (NASDA) include developing aquatic animal experiment facilities for NASA space shuttle missions, yielding valuable insights into life support systems and in-flight research protocols [4].

Building on these international advancements, China has enhanced its space life science capabilities through the completion of the China Space Station (CSS), which offers a long-term, stable orbital platform for investigating animal physiological and behavioral responses to microgravity, high radiation, and weak magnetic fields [5,6,7]. Among model organisms used in such research, *Drosophila* and zebrafish are particularly valuable due to their genetic homology with humans, short life cycles, and well-characterized behavioral repertoires—traits that facilitate elucidation of fundamental biological adaptation mechanisms in space [8]. Zebrafish specifically have emerged as a key species for testing integrated life support systems in long-duration missions, where development of biological life support systems is imperative. This context frames the 43-day closed aquatic ecosystem experiment aboard the CSS, which successfully maintained carefully selected zebrafish (screened for behavioral, physiological, and social compatibility) alongside Ceratophyllum and microorganisms, achieving the main scientific objectives [9].

Advances in experimental methodologies have allowed high-resolution imaging systems aboard the CSS to generate massive video datasets, capturing detailed behavioral dynamics of animals over extended periods. These rich behavioral datasets require automated, accurate, and robust tracking tools to circumvent the limitations of traditional manual or contact-based observation methods, which are labor-intensive and impractical in space environments [10,11,12].

Tracking animals in space environments presents unique and largely unaddressed challenges. The synergistic effects of microgravity, radiation, and altered magnetic fields induce erratic and unpredictable movement patterns, posing significant obstacles to conventional tracking algorithms [13,14]. Three key challenges are particularly prominent: first, animals within the same culture unit often exhibit high appearance similarity due to synchronized development, complicating individual differentiation—especially during trajectory crossings; second, microgravity induces complex motion patterns (e.g., sudden accelerations and sharp turns) that exceed the modeling capacity of traditional motion-based tracking approaches; and third, dense group interactions cause frequent occlusions, leading to trajectory fragmentation and identity switches in tracking outputs.

To address these challenges, this study proposes a novel deep learning-based MOT framework specifically designed for the unique scenarios of space animal tracking, including microgravity-induced erratic motion, high appearance similarity, and frequent occlusions. The framework integrates a heterogeneous graph neural network architecture with decoupled appearance–motion modeling and a cross-modal re-identification mechanism.

The proposed framework is evaluated using the publicly available SpaceAnimals Dataset, which comprises space-observed *Drosophila* and zebrafish captured under real space environmental conditions. Experimental results demonstrate that this method significantly outperforms state-of-the-art MOT approaches in both tracking accuracy and robustness. The key contributions of this study are as follows:

- **A multi-modal feature fusion framework:** A deep learning architecture is proposed that separates and integrates appearance and motion features of space animals via a heterogeneous graph network, enhancing MOT performance in extreme space environments.
- **A motion decoupling method:** A local polynomial approximation method is introduced to decompose motion components, enabling accurate estimation of speed and acceleration and improving tracking robustness for space animals under microgravity.
- **A cross-modal re-detection module:** A cross-modal re-detection method is designed to align appearance and motion features for identity maintenance, facilitating recovery of lost tracks during occlusions or rapid movements of space animals.

This work underscores the potential of artificial intelligence (AI) as a powerful tool in animal behavioral studies, providing reliable solutions for space animal tracking and behavior analysis in orbital and deep-space environments. It further opens new avenues for future applications in space life sciences and the investigation of animal behavioral genetics.

## 2. Related Work

Recent advancements in AI and computer vision have significantly enhanced MOT, facilitating accurate and non-intrusive behavioral analysis in complex environments [15]. MOT methods are primarily categorized into detection-based tracking (DBT) and joint detection and tracking (JDT). DBT leverages object detectors such as YOLO [16,17] or Faster R-CNN [18], followed by association strategies based on appearance or motion [19]. However, appearance-based methods are susceptible to occlusions and lighting variations, while motion-based approaches often fail under abrupt movements [20].

JDT methods integrate detection and tracking within a unified framework. For instance, Transformer-based models like TransTrack [21] enable simultaneous detection and tracking via global attention, albeit with high computational overhead. FairMOT [22], built on CenterNet [23], enhances identity preservation in crowded scenes by incorporating Re-ID features into the detection process.

Despite substantial progress in MOT, existing algorithms encounter notable challenges when applied to animal behavioral analysis. Specifically, many animal species exhibit minimal inter-individual visual differences, rendering appearance-based tracking methods prone to identity mismatches and tracking errors [24,25,26]. To address this limitation, researchers have increasingly adopted motion-based tracking approaches [27,28,29].

Manoukis et al. [30] introduced a constant-velocity Markov process augmented with stochastic perturbations to improve tracking accuracy in densely populated animal groups. Angarita [31] enhanced tracking continuity by incorporating a global temporal smoothness optimization strategy. Wu et al. [32] addressed one-to-one matching constraints through a combination of particle filtering and multi-view tracking. Wang et al. [33] further advanced this line of research by integrating long short-term memory (LSTM) networks with particle filtering to more effectively model animal movement dynamics. Recently, Yin et al. [34] demonstrated the potential of Transformer-based architectures in capturing intricate motion patterns within animal groups, highlighting the efficacy of attention mechanisms for long-term tracking in complex environments. WildLive [35] enables real-time, onboard animal tracking in drones through optimized YOLO detection and sparse optical flow, achieving 17.8 fps. Tested on over 200K annotated wildlife videos, the system outperforms existing methods and supports autonomous wildlife monitoring. Tang et al. [36] introduced the anti-drift pose tracker (ADPT), a Transformer-based method that significantly reduces drift and outperforms existing tools in accuracy, achieving up to 99.72% identity accuracy in social mouse interactions while enabling efficient, real-time, end-to-end analysis.

## 3. Method

**Figure 1:**
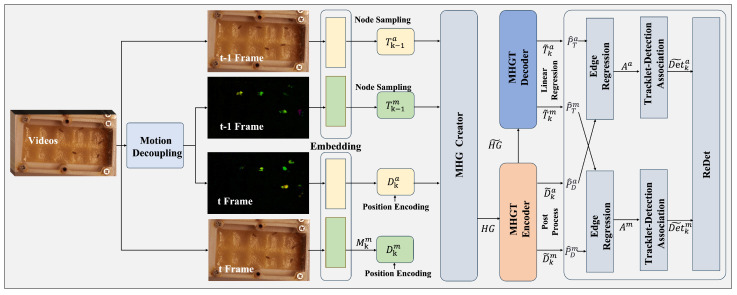
Framework of the proposed MOT method for space animals.

This paper presents an MOT framework based on a Multi-modal Heterogeneous Graph Transformer (MHGT) for space animals. As illustrated in Figure 1, the framework fuses spatio-temporal information from appearance and motion features to enable joint detection and tracking, comprising three core components: motion decoupling, cross-modal feature fusion, and a unified detection–tracking module.

### 3.1. Motion Decoupling

The proposed motion decoupling framework is designed to effectively separate meaningful motion patterns from noise and artifacts in space animal tracking scenarios. It adopts a three-stage hierarchical architecture: motion modeling, polar transformation with adaptive thresholding, and dual-threshold filtering. These stages operate sequentially to extract reliable motion features for subsequent multi-modal fusion.

The first stage models local motion via second-order Taylor expansion to approximate intensity variations within small spatial neighborhoods. Specifically, for a pixel location (x,y), the image intensity is expressed as:

(1) $$I\left(x,y\right)=\frac{1}{2}\left[\begin{array}{cc} x & y \end{array}\right]\left[\begin{array}{cc} A_{xx} & A_{xy}\\ A_{xy} & A_{yy} \end{array}\right]\left[\begin{array}{c} x\\ y \end{array}\right]+\left[\begin{array}{cc} b_{x} & b_{y} \end{array}\right]\left[\begin{array}{c} x\\ y \end{array}\right]+c$$

where $A\in R^{2\times 2}$ denotes the Hessian matrix capturing local curvature, $b\in R^{2}$ represents the gradient vector, and *c* is the intensity at the center. For two consecutive frames $I_{t}$ and $I_{t+\Delta t}$, the displacement (Δx,Δy) is estimated by solving the regularized optimization problem.

(2) $$\min_{A,b,c}\sum_{(x,y)\in \Omega }{∥I_{t}\left(x,y\right)-I_{t+\Delta t}\left(x+\Delta x,y+\Delta y\right)∥}_{2}^{2}+\lambda \left(\alpha {∥A∥}_{F}^{2}+\beta {∥b∥}_{2}^{2}\right)$$

where Ω denotes a 5×5 local window and α=0.1 and β=0.01 are empirically determined to mitigate overfitting.

The resulting displacement vectors are transformed into polar coordinates to enhance the capture of motion magnitude and direction.

(3) $$\left\{\begin{array}{c} \rho =\sqrt{\Delta x^{2}+\Delta y^{2}}\\ \theta =\arctan2(\Delta y,\Delta x) \end{array}\right.$$

Local statistics of the motion magnitude ρ are computed within sliding windows to derive the mean $\mu_{\rho }$ and standard deviation $\sigma_{\rho }$. Based on these statistics, adaptive thresholds are defined as:

(4) $$\tau_{\mathrm{low}}=\mu_{\rho }+k_{1}\sigma_{\rho },\;\tau_{\mathrm{high}}=\mu_{\rho }+k_{2}\sigma_{\rho }$$

with $k_{1}=1.0$ and $k_{2}=2.5$ being selected to effectively differentiate between noise and actual motion.

In the final filtering stage, each pixel is categorized into one of three classes based on its motion magnitude.

(5) $$M\left(x,y\right)=\left\{\begin{array}{cc} 0 & \mathrm{if}\;\rho \left(x,y\right)<\tau_{\mathrm{low}}\\ 1 & \mathrm{if}\;\tau_{\mathrm{low}}\leq \rho \left(x,y\right)\leq \tau_{\mathrm{high}}\\ 2 & \mathrm{if}\;\rho \left(x,y\right)>\tau_{\mathrm{high}} \end{array}\right.$$

Here, M(x,y)=0 suppresses static background regions, M(x,y)=1 marks uncertain motion, and M(x,y)=2 indicates confident motion. The final motion features are retained as:

(6) $$F_{\mathrm{motion}}=\left\{\left(\rho \left(x,y\right),\theta \left(x,y\right)\right)|M\left(x,y\right)>0\right\}$$

which forms a sparse yet informative representation. This reduces computational overhead significantly while preserving essential motion cues for appearance–motion fusion.

### 3.2. Cross-Modal Feature Fusion

To effectively integrate appearance and motion information in space animal tracking, this study proposes a cross-modal feature fusion framework based on a heterogeneous graph network [37]. The framework employs a four-stage pipeline: feature extraction, graph construction, attention-based message passing, and deformable decoding. Each stage is specifically designed to progressively model complex inter- and intra-modality relationships.

The process initiates with multi-scale feature extraction via the PVTv2 [38] backbone, which processes each input frame through four hierarchical stages with downsampling ratios r∈{4,8,16,32}. At each stage *s*, pyramidal features $M_{k}^{a/m,s}$ are derived for both appearance and motion modalities. These 3D feature maps are flattened and linearly projected into 2D query matrices as:

(7) $$D_{k}^{a/m,s}=\mathrm{Flatten}\left(M_{k}^{a/m,s}\right)W_{p}^{s}+p^{s}$$

where $W_{p}^{s}$ denotes learnable projection weights and $p^{s}$ represents positional encodings. The latter are computed using sinusoidal functions across L=8 multi-scale frequency bands to capture spatial context.

Based on the extracted features, this paper proposes a Multi-Modal Heterogeneous Graph Network (MHGN), denoted as $G_{k}=\left(V_{k},E_{k}\right)$. Here, $V_{k}$ is the node set defined as $V_{k}=\left\{D_{k}^{a},D_{k}^{m},T_{k-1}^{a},T_{k-1}^{m}\right\}$, where $D_{k}^{a}$ and $D_{k}^{m}$ correspond to the appearance and motion queries of the current frame, while $T_{k-1}^{a}$ and $T_{k-1}^{m}$ denote the trajectory memory from the previous frame (initialized to zero). The edge set $E_{k}=\left\{E_{t},E_{s},E_{h}\right\}$ comprises three edge types: $E_{t}$ (temporal edges), connecting $D_{k}^{a/m}$ and $T_{k-1}^{a/m}$ to model temporal consistency; $E_{s}$ (spatial edges), capturing spatial proximity via relative positional bias; and $E_{h}$ (heterogeneous edges), linking $D_{k}^{a}$ and $D_{k}^{m}$ to enable cross-modal interaction.

**Figure 2:**
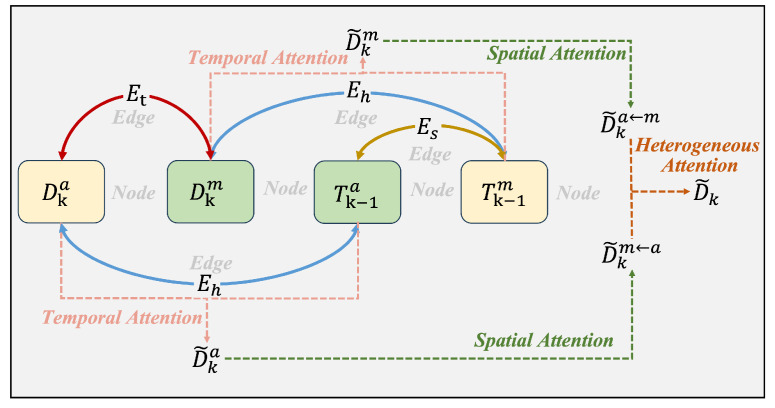
The topological structure of the proposed heterogeneous graph network.

**Figure 3:**
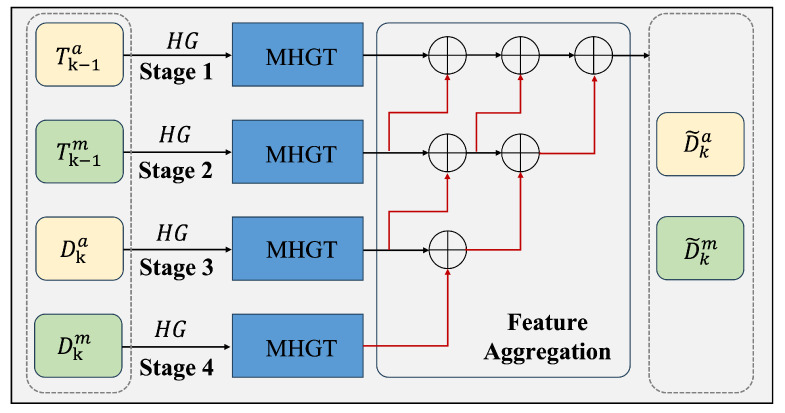
The structure of the MHGN encoding.

**Figure 4:**
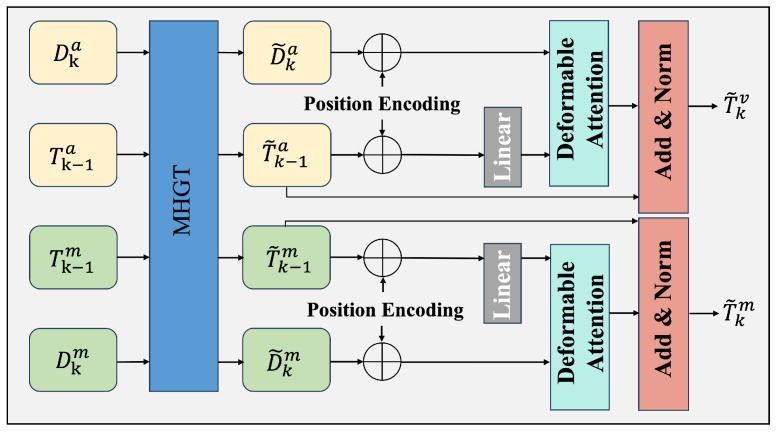
The structure of the MHGN decoding.

In the attention encoding stage, context-aware query representations are generated by processing this graph structure through three specialized attention mechanisms: temporal attention, spatial attention, and heterogeneous attention. The topological structure of the proposed heterogeneous graph network is illustrated in Figure 2. As shown in Figure 3 and Figure 4, the encoder models spatio-temporal dependencies and learns inter-modality mappings via modality-specific attention. Meanwhile, the feature fusion process described in Algorithm 1 outlines the key steps of the model selection pipeline.

First, modality-specific temporal attention updates node features via intra-modal self-attention:

(8) $${\mathrm{Attn}}_{t}\left(Q,K,V\right)=\mathrm{Softmax}\left(\frac{QW_{Q}^{t}{\left(KW_{K}^{t}\right)}^{⊤}}{\sqrt{d}}\right)VW_{V}^{t}$$

where $W_{Q}^{t},W_{K}^{t},W_{V}^{t}$ denote modality-specific parameters.

Second, cross-modality spatial attention captures inter-modal spatial dependencies using relative position bias:

(9) $$\mathrm{Attn}s\left(Q,K,V\right)=\mathrm{Softmax}\left(\frac{QW_{Q}^{s}{\left(KW_{K}^{s}\right)}^{⊤}}{\sqrt{d}}+\phi ij\right)VW_{V}^{s}$$

with $\phi_{ij}=\mathrm{MLP}\left(\Delta x_{ij},\Delta y_{ij}\right)$ encoding relative spatial offsets.

Third, heterogeneous relation modeling aggregates information across distinct node types:

(10) $$\mathrm{Attn}h\left(Q,K,V\right)=\sum m\in a,m\mathrm{Softmax}\left(\frac{QW_{Q}^{h}{\left(K_{m}W_{K}^{h}\right)}^{⊤}}{\sqrt{d}}\right)V_{m}W_{V}^{h}$$

This multi-level attention framework enables the model to learn enriched cross-modal representations while preserving modality-specific attributes. The refined features are subsequently fed into a deformable attention decoder for final query updating:

(11) $$\tilde{T}k=\sum {m=1}^{M}W_{m}\left[\sum_{q\in Rm}Amq\cdot \tilde{D}k\left(x_{q}+\Delta xmq,y_{q}+\Delta y_{mq}\right)\right]$$

where M=8 denotes the number of attention heads, $R_{m}$ represents reference points, $\left(\Delta x_{mq},\Delta y_{mq}\right)$ are learnable offsets, and $A_{mq}$ corresponds to attention weights.
**Algorithm 1** Cross-modal feature fusion
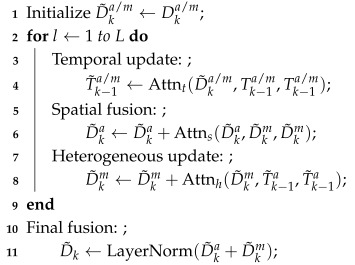


The integrated fusion framework thereby achieves robust integration of appearance and motion cues, facilitating accurate and context-aware tracking of animals in complex spatial environments.

### 3.3. Unified Detection–Tracking Framework

The proposed framework seamlessly integrates object detection and tracking into a single end-to-end trainable architecture, enabling real-time, accurate, and robust animal tracking in spatial environments. Detection and tracking operate in parallel using shared multi-modal features: detection outputs provide initial object hypotheses, while tracking queries maintain temporal continuity across frames.

The detection head generates object predictions from fused multi-modal features ${\tilde{D}}_{k}^{a/m}\in R^{N_{d}\times h}$. These features are first reshaped and processed via a series of convolutional layers to produce three key outputs: a center heatmap $H_{k}$ (with sigmoid activation) indicating object presence, bounding box coordinates $B_{k}=\left(x,y,w,h\right)$, and refinement offsets $O_{k}$ (with Tanh activation) for precise localization. The detection branch is formulated as follows.

(12) $$\left\{\begin{array}{c} H_{k}=\sigma \left({\mathrm{Conv}}_{3\times 3}\left(\mathrm{ReLU}\left({\mathrm{Conv}}_{1\times 1}\left({\tilde{D}}_{k}^{a/m}\right)\right)\right)\right)\\ B_{k}={\mathrm{Conv}}_{1\times 1}\left(\mathrm{ReLU}\left({\mathrm{Conv}}_{3\times 3}\left({\tilde{D}}_{k}^{a/m}\right)\right)\right)\\ O_{k}=\mathrm{Tan}h\left({\mathrm{Conv}}_{1\times 1}\left(\mathrm{ReLU}\left({\mathrm{Conv}}_{3\times 3}\left({\tilde{D}}_{k}^{a/m}\right)\right)\right)\right) \end{array}\right.$$

For tracking, a set of learned tracking queries $\tilde{T}\in R^{N_{t}\times h}$ encodes historical object states. These queries are processed through a displacement prediction network implemented as a multi-layer perceptron:

(13) $$\Delta_{k|k-1}=W_{2}\mathrm{ReLU}\left(W_{1}\tilde{T}+b_{1}\right)+b_{2}$$

where $W_{1}\in R^{h\times 2h}$ and $W_{2}\in R^{2h\times 2}$ denote learnable weights. Updated object positions are estimated as:

(14) $$p_{k}=p_{k-1}+\Delta_{k|k-1}+\epsilon_{k}$$

with $\epsilon_{k}∼N\left(0,\Sigma \right)$ modeling motion uncertainty.

To associate newly detected objects with existing tracks, a cross-frame affinity matrix $A\in R^{N_{d}\times N_{t}}$ is computed to measure similarity between detection and tracking queries. The affinity score between detection $U_{i}$ and track $V_{j}$ is derived using a learned scoring function:

(15) $$Aij=\frac{1}{1+\exp(-sij)},\;s_{ij}=f_{\theta }\left(U_{i},V_{j}\right)$$

where $f_{\theta }$ is defined as:

(16) $$f_{\theta }\left(U,V\right)=W_{2}^{⊤}\mathrm{ReLU}(W_{1}\left[U;V;U\circ V;|U-V|\right]+b_{1})+b_{2}.$$

This affinity matrix is subsequently used to solve the bipartite matching problem via the Hungarian algorithm:

(17) $$\hat{M}=\arg\max_{M\in P(N_{d},N_{t})}\sum_{(i,j)\in M}A_{ij}.$$

To enhance detection reliability, a cross-modal confidence fusion strategy is introduced to leverage the complementary strengths of appearance and motion cues. The refined detection confidence is calculated as:

(18) $$\tilde{Det}k^{i}=\frac{Det_{k}^{i}}{1+\exp(-\gamma c{k,i}^{h})},\;\gamma =2.0,$$

where $c_{k,i}^{h}$ represents the harmonic mean of modality-specific confidences:

(19) $$c_{k,i}^{h}=\frac{2c_{k}^{a}c_{k}^{m}}{c_{k}^{a}+c_{k}^{m}+\epsilon }$$

The entire framework is trained using a multi-task loss function that jointly optimizes detection, tracking, and association:

(20) $$L=L_{cf}+L_{bs}+L_{r}+L_{td}+L_{match}$$

where $L_{cf}$ denotes the center-focused loss for penalizing heatmap prediction errors, $L_{bs}$ quantifies bounding box regression accuracy via SmoothL1 loss, $L_{r}$ ensures precise refinement of object centers, $L_{td}$ aligns predicted and ground truth motion displacements using IoU, and $L_{match}$ optimizes the data association matrix through binary cross-entropy.

This unified design enables the model to maintain consistent long-term object identities while adapting to dynamic and challenging visual conditions in spatial animal tracking scenarios.

## 4. Experiments

### 4.1. Experimental Data

This study utilizes the SpaceAnimal Dataset [39], the first publicly available dataset specifically developed for the non-contact behavioral analysis of multiple animal species in space environments. The dataset includes two model organisms with well-characterized cognitive behaviors: *Drosophila* and zebrafish, selected as experimental subjects to validate the proposed method. The *Drosophila* cohort was transported to the CSS via the Tianzhou-8 cargo spacecraft on 15 November 2024, successfully completing three generations under microgravity conditions. The zebrafish cohort was delivered to the CSS earlier by the Shenzhou-18 manned mission on 25 April 2024, achieving a breakthrough in on-orbit spawning.

High-resolution video data were collected throughout the life cycles of both species. Researchers selected representative time periods, performed detailed manual annotations, and constructed a multi-object tracking dataset. The *Drosophila* subset contains 20 video segments, totaling 2500 image frames and 27,500 individual instances, while the zebrafish subset comprises 8 video segments, with 1757 image frames and 7028 individual instances.

The dataset was partitioned into training, validation, and test subsets using a temporal split strategy to prevent data leakage and ensure realistic evaluation of model generalization. Specifically, the split ratio was 60% for training, 10% for validation, and 30% for testing, based on distinct experimental sessions. To avoid temporal overlap, all videos from the same session were assigned to the same subset. The training set was used for model learning, the validation set for hyperparameter tuning and early stopping, and the test set exclusively for final evaluation without involvement in the training process.

### 4.2. Metrics

This paper evaluates tracking performance using widely adopted MOT metrics, including multiple object tracking accuracy (MOTA) [40] and identification F1 score (IDF1) [41], which are defined as follows:

(21) $$\mathrm{MOTA}=1-\frac{\sum_{t}\left({\mathrm{FP}}_{t}+{\mathrm{FN}}_{t}+{\mathrm{IDSW}}_{t}\right)}{\sum_{t}{\mathrm{GT}}_{t}}$$

(22) $$\mathrm{IDF}1=\frac{2\mathrm{IDTP}}{2\mathrm{IDTP}+\mathrm{IDFP}+\mathrm{IDFN}}$$

where MOTA quantifies overall tracking performance by aggregating frame-wise errors—including false positives (FPs), false negatives (FNs), and identity switches (IDSWs)—normalized by the total number of ground truth objects (GTs). Higher MOTA scores indicate better alignment between predicted and true trajectories.

IDF1 evaluates the accuracy of identity preservation over time, computed using ID true positives (IDTPs), ID false positives (IDFPs), and ID false negatives (IDFNs). Values closer to 1 denote stronger consistency in individual object identification across frames.

Additionally, we report trajectory fragmentation (Frag), which counts the total number of trajectory interruptions during tracking. Lower Frag values indicate that the tracker maintains more continuous and stable trajectories, a critical attribute for long-term behavioral analysis.

### 4.3. Implementation Details

All experiments were conducted using PyTorch on an Ubuntu 18.04 OS. The machine was equipped with an Intel(R) Core(TM) i9 CPU @ 3.30 GHz, 128 GB of memory, and 2× GTX 3090 GPUs.

The model was trained with the following specifications (Table 1):

## 5. Results

### 5.1. Comparison with State-of-the-Art Methods

Table 2 presents comparative results on the *SpaceAnimal Dataset*, where our method is evaluated against six state-of-the-art tracking approaches: CenterTrack [42], TransCenter [43], TrackFormer [44], ByteTrack [45], MOTRv2 [46], and Hybrid-SORT [47]. The assessment spans two model species (*Drosophila* and zebrafish) using three metrics: MOTA, IDF1, and Frag (trajectory fragmentation).

In the *Drosophila* subset, our method achieves MOTA = 88.21%, IDF1 = 85.06%, and Frag = 42, outperforming all comparative methods. It surpasses ByteTrack—the second-highest performer in MOTA (75.21%)—by +13.0%, indicating substantial improvements in handling high-density, fast-moving targets under microgravity. TrackFormer exhibits the lowest MOTA (67.48%), likely due to its limited capability for motion modeling in dynamic environments.

For identity preservation, our method outperforms the second-ranked MOTRv2 (75.35%) by +9.71% in IDF1, demonstrating the efficacy of our cross-modal re-detection mechanism in maintaining identity consistency during occlusions and rapid movements. Hybrid-SORT and TrackFormer show notably lower IDF1 scores (66.23% and 66.25%, respectively), indicating deficiencies in long-term identity maintenance. Our method also achieves the lowest trajectory fragmentation (Frag = 42), significantly outperforming ByteTrack (91) and CenterTrack (98), which highlights its ability to sustain continuous, stable tracking of small, fast-moving targets.

Among comparative methods, ByteTrack performs relatively well in both MOTA and Frag but exhibits a substantially lower IDF1 score (76.50%) compared with our method, indicating limitations in temporal identity association. CenterTrack follows a similar pattern, with strong detection performance but weaker identity management.

In the zebrafish subset, our method again achieves superior performance with MOTA = 82.21%, IDF1 = 74.26%, and Frag = 36. It outperforms MOTRv2—the second-highest performer in MOTA (78.14%)—by +4.07%, demonstrating robustness in handling larger, more complex motion patterns typical of aquatic organisms. TransCenter exhibits the lowest MOTA (60.20%), likely due to its sensitivity to appearance degradation under low-contrast or motion-blurred conditions.

Our method also attains the highest IDF1 score (74.26%), surpassing MOTRv2 (64.24%) by +10.02%. This highlights its superiority in distinguishing visually similar individuals and maintaining identity across long sequences—a critical requirement for group behavior analysis. CenterTrack and ByteTrack show comparable IDF1 scores (60.14% and 62.95%, respectively), indicating moderate identity consistency but remaining inferior to our method.

For trajectory continuity, our method achieves the lowest Frag = 36, significantly outperforming MOTRv2 (78) and CenterTrack (85). This underscores its ability to generate smooth, uninterrupted trajectories—essential for capturing fine-grained behavioral patterns such as schooling and social interaction.

Among comparative methods, MOTRv2 performs best on zebrafish, particularly in MOTA and Frag, but its lower IDF1 score indicates limitations in identity preservation. CenterTrack and ByteTrack offer balanced performance but are outperformed by our method across all three metrics.

Across both species, several key trends emerge. ByteTrack and CenterTrack provide strong baseline performance in MOTA and Frag but struggle with identity preservation, especially in long-term tracking. MOTRv2 excels on zebrafish, likely due to its motion modeling capabilities, but underperforms on *Drosophila*, suggesting limited adaptability to smaller, faster-moving targets. TrackFormer and TransCenter show consistently lower performance across all metrics, indicating that their Transformer-based architectures may be ill-suited to the unique visual and motion characteristics of spatial animal tracking. Hybrid-SORT, which integrates detection with Kalman filtering, exhibits moderate performance but fails to match our method in identity consistency and trajectory smoothness.

**Figure 5:**
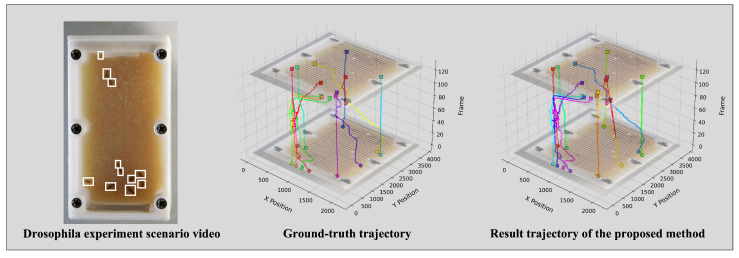
Visualization comparison of tracking results of *Drosophila*.

**Figure 6:**
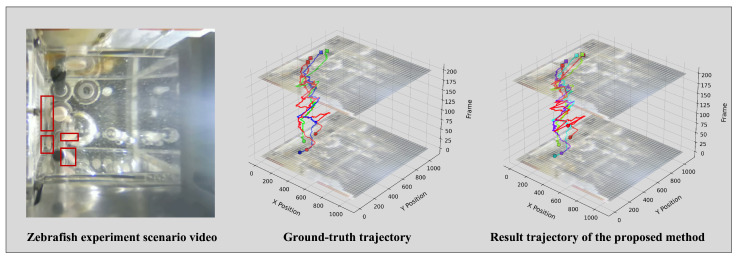
Visualization comparison of tracking results of zebrafish.

Figure 5 and Figure 6 compare ground truth trajectories with the tracking results of the proposed method for *Drosophila* and zebrafish under microgravity. Despite complex motion patterns, predicted trajectories exhibit high consistency with true paths, confirming accurate tracking performance.

In summary, our method outperforms comparative approaches across all metrics and both species, demonstrating superior tracking accuracy, stronger identity consistency, and more stable trajectory estimation. These improvements are particularly pronounced in microgravity environments, where irregular motion patterns and suboptimal visual conditions are explicitly addressed by our architecture through a heterogeneous graph network and cross-modal identity re-detection mechanism.

We report the average inference speed, model size, and FLOPs of our framework on the SpaceAnimal Dataset using two NVIDIA RTX GPUs: an inference speed of 28.5 FPS (frames per second) for 800×800 resolution videos, a model size of 53.4 MB, and 18.7 GFLOPs per frame. These metrics confirm that our method achieves real-time performance with reasonable computational requirements, making it suitable for long-term behavioral monitoring in resource-constrained environments such as space stations.

### 5.2. Ablation Study

We conducted an ablation study to evaluate the contribution of each proposed component—motion decomposition (Motion), Multi-Modal Heterogeneous Graph Network (MHGN), and cross-modal re-detection (ReDet)—on the *Drosophila* and zebrafish subsets of the SpaceAnimal Dataset. The results are summarized in Table 3, where the baseline refers to the core tracking framework without any additional modules. Components are incrementally introduced, with performance evaluated using MOTA, IDF1, and the MT/ML metric (which quantifies tracking performance by comparing the count of consistently tracked targets [MT: ≥ 80% lifespan coverage] against frequently lost targets [ML: ≤ 20% lifespan coverage]).

The baseline achieves MOTA = 74.41% and IDF1 = 79.59% on *Drosophila* and MOTA = 74.12% and IDF1 = 60.14% on zebrafish. While detection performance is acceptable, identity consistency and robustness are limited—particularly under complex microgravity motion—as indicated by moderate MT/ML ratios (61/12 and 58/15) and frequent identity switches.

Adding motion decomposition improves both tracking accuracy and identity preservation. On *Drosophila*, MOTA increases by +7.22% to 81.63%, and IDF1 rises by +2.86% to 82.45%. On zebrafish, improvements are more pronounced: MOTA = 79.09% (+4.97%) and IDF1 = 67.79% (+7.65%). MT/ML ratios also improve to 73/8 and 65/11, reflecting fewer track losses and more complete trajectories. These results demonstrate that motion decomposition enhances motion estimation and tracking stability in dynamic environments.

Integrating MHGN further improves performance by fusing appearance and motion features. On *Drosophila*, MOTA = 86.45% (+4.82%) and IDF1 = 84.36% (+1.91%); on zebrafish, MOTA = 80.74% (+1.65%) and IDF1 = 71.22% (+3.43%). MT/ML ratios reach 82/5 and 72/8, indicating stronger association capability and fewer lost tracks. These findings confirm that MHGN enhances feature representation and robustness under occlusion and visual ambiguity.

Finally, introducing ReDet to maintain identity and recover lost tracks during occlusions or fast motion yields further gains: on *Drosophila*, MOTA = 88.21% (+1.76%) and IDF1 = 85.06% (+0.70%); on zebrafish, MOTA = 82.21% (+1.47%) and IDF1 = 74.26% (+3.04%). MT/ML ratios reach 86/4 and 78/6, demonstrating that ReDet effectively mitigates identity drift and track loss.

Across both species, each component contributes to performance improvement in a distinct, complementary manner. Motion decomposition enhances motion modeling and trajectory estimation, particularly in dynamic, unpredictable environments. MHGN strengthens feature fusion and association robustness by integrating multi-modal cues within a graph-based structure. ReDet ensures identity consistency and improves re-identification capability, especially during occlusions or rapid movements.

Together, these modules form a cohesive architecture that significantly outperforms the baseline and achieves state-of-the-art performance on the SpaceAnimal Dataset. The ablation results provide strong evidence that the design choices are well motivated and effective in addressing the unique challenges of animal tracking in spatial environments.

## 6. Discussion

### 6.1. Key Findings

This study presents a novel deep learning-based MOT framework tailored for tracking animals in extreme space environments, addressing the unique challenges posed by microgravity, high radiation, and weak magnetic fields. The experimental results on the SpaceAnimal Dataset—encompassing *Drosophila* and zebrafish—demonstrate that our method outperforms state-of-the-art MOT approaches across critical metrics, including MOTA, IDF1, and Frag.

The ablation study further confirms the contributions of individual components: motion decomposition enhances trajectory stability by accurately estimating speed and acceleration under microgravity; MHGN strengthens feature representation by leveraging inter-modal dependencies; and the cross-modal re-detection module mitigates identity switches during occlusions or rapid movements. Together, these components form a cohesive architecture that balances accuracy and robustness, addressing the limitations of existing methods in handling space-specific challenges.

### 6.2. Limitations

The proposed method, while effective in tracking *Drosophila* and zebrafish under space conditions, has limitations that restrict its broader applicability. Scalability to larger animal models is a key challenge, as the current design is optimized for small organisms with relatively simple locomotor patterns. Larger animals like rodents exhibit more complex behaviors, including diverse postures and interactions, which may strain the motion decoupling module and cross-modal fusion mechanisms, with non-rigid motion potentially being insufficiently modeled by the current local polynomial approximation-based motion decomposition. Additionally, the method relies on high-quality input, degrading under severe motion blur, low lighting, or occlusions, and assumes a static camera setup, struggling with viewpoint changes or dynamic backgrounds.

Hardware and computational constraints further limit deployment, as the method, despite achieving 28.5 FPS on 800×800 resolution, requires significant resources like dual GTX 3090 GPUs, which may not align with spacecraft power and weight limitations. The heterogeneous graph network’s computational cost, especially during attention encoding, hinders real-time use in resource-constrained scenarios, and prolonged occlusions or highly similar animal appearance and motion patterns can cause identity switches.

## 7. Conclusions

To address the challenges of multi-object tracking for spatial animals in microgravity environments, this paper proposes a novel MOT method. The framework integrates a motion decoupling module to extract motion components (e.g., velocity and acceleration), which—along with appearance features—serve as dual-input modalities. These features are encoded and fused via a heterogeneous graph network, which effectively integrates cross-modal spatiotemporal information to enhance feature representation. A cross-modal re-detection module is further introduced to maintain identity consistency and recover lost tracks during occlusions or fast movements.

Experimental results on the SpaceAnimal Dataset demonstrate that the proposed method achieves state-of-the-art performance, significantly outperforming existing approaches in key tracking metrics (including MOTA and IDF1). The ablation study confirms the effectiveness of each component in improving tracking accuracy, robustness, and identity preservation. For future work, we aim to generalize the framework to a broader range of multi-object tracking tasks, evaluating its adaptability and performance across diverse application scenarios.

## Figures and Tables

**Table 1:** Training hyperparameters.

Parameter	Value
Optimizer	AdamW
Initial Learning Rate	2 × $10^{-4}$
Batch Size	16
Training Epochs	300
Learning Rate Schedule	Cosine Annealing
Warmup Steps	1000
Weight Decay	0.05
Input Resolution	512 × 512
Augmentation	Random Flip, Rotation (±$30^{\circ }$)

**Table 2:** Performance benchmarking of MOT algorithms for space animals.

Method	*Drosophila*			Zebrafish		
	MOTA ↑	IDF1 ↑	Frag ↓	MOTA ↑	IDF1 ↑	Frag ↓
CenterTrack [42]	74.41%	79.59%	98	74.12%	60.14%	85
TransCenter [43]	72.58%	74.12%	113	60.20%	63.13%	102
TrackFormer [44]	67.48%	66.25%	96	58.26%	59.13%	115
ByteTrack [45]	75.21%	76.50%	91	75.90%	62.95%	82
MOTRv2 [46]	61.93%	75.35%	125	78.14%	64.24%	78
Hybrid-SORT [47]	70.62%	66.23%	98	72.34%	61.25%	91
**Ours**	**88.21%**	**85.06%**	**42**	**82.21%**	**74.26%**	**36**

**Table 3:** Component ablation study.

Configuration	*Drosophila*			Zebrafish		
	MOTA ↑	IDF1 ↑	MT ↑/ML ↓	MOTA ↑	IDF1 ↑	MT ↑/ML ↓
Baseline	74.41%	79.59%	61/12	74.12%	60.14%	58/15
+ Motion	81.63%	82.45%	73/8	79.09%	67.79%	65/11
++ MHGN	86.45%	84.36%	82/5	80.74%	71.22%	72/8
+++ ReDet	88.21%	85.06%	86/4	82.21%	74.26%	78/6

## Data Availability

The SpaceAnimals datasets presented in this study can be accessed at the following link: https://doi.org/10.1038/s41597-025-05111-8.
